# Neural substrates of early executive function development

**DOI:** 10.1016/j.dr.2019.100866

**Published:** 2019-06

**Authors:** Abigail Fiske, Karla Holmboe

**Affiliations:** a School of Psychology, University of Nottingham, Nottingham, United Kingdom; b Department of Experimental Psychology, University of Oxford, Oxford, United Kingdom

**Keywords:** Executive function, Prefrontal cortex, Brain connectivity, Development, Infancy, Childhood, Neuroimaging

## Abstract

In the last decade, advances in neuroimaging technologies have given rise to a large number of research studies that investigate the neural underpinnings of executive function (EF). EF has long been associated with the prefrontal cortex (PFC) and involves both a unified, general element, as well as the distinct, separable elements of working memory, inhibitory control and set shifting. We will highlight the value of utilising advances in neuroimaging techniques to uncover answers to some of the most pressing questions in the field of early EF development. First, this review will explore the development and neural substrates of each element of EF. Second, the structural, anatomical and biochemical changes that occur in the PFC during infancy and throughout childhood will be examined, in order to address the importance of these changes for the development of EF. Third, the importance of connectivity between regions of the PFC and other brain areas in EF development is reviewed. Finally, throughout this review more recent developments in neuroimaging techniques will be addressed, alongside the implications for further elucidating the neural substrates of early EF development in the future.

## Highlights

• Advances in neuroimaging have facilitated the study of EF development early in life.
• Recent research continues to identify the pivotal role of the PFC in EF.
• Important changes occur in the prefrontal cortex across development.
• The importance of brain connectivity for EF is becoming increasingly clear.
• Functional brain specialisation for EF continues to develop into adolescence.

## Introduction

The development of executive function (EF) from infancy to an age of cognitive maturity is a widely studied area of great interest to developmental psychologists. Working memory (WM), inhibitory control (IC) and set shifting/cognitive flexibility are three core components that constitute the suite of cognitive processes commonly referred to as executive function. Although to some extent separable, these components share a common purpose: the allocation of attention and control over behaviour, in order to meet an adaptive goal (Friedman and Miyake, 2017, Miyake and Friedman, 2012, Miyake et al., 2000). The developmental trajectories and neural substrates of EF have been extensively researched in recent years, spurred by the increased use of neuroimaging techniques among developmental psychologists (See Box 1).

One of the first comprehensive reviews of the development of EF from infancy to early adulthood was published by Adele Diamond in 2002. Diamond’s chapter discussed the normal maturation of the prefrontal cortex (PFC) by integrating evidence of developments in cognitive functioning, anatomy and biochemistry. However, Diamond’s chapter closed with a wealth of unanswered questions that were ripe for future investigation at the time: “what are the developmental changes in the PFC that underpin the improvement in cognitive functions?”, “what role do regions beyond the dorsolateral PFC play in subserving cognitive functions?”, and “when do functional connections between frontal regions develop?” (Diamond, 2002, p. 494). Indeed, Diamond (2002, p. 495) concluded by recognising that the key to answering many of these questions may lie in the “tools” that are now available to researchers. From these closing remarks (Diamond, 2002), we have therefore identified four broad themes that we will seek to address through this current review: Neural substrates of components of EF, PFC development, frontal connectivity, and advances in neuroimaging. Thus, with this paper, we aim to provide an updated literature review that incorporates findings from modern neuroimaging studies in order to provide insight into some of the answers Diamond was seeking in 2002.

Box 1 **Traditional and new neuroimaging techniques in developmental research**

Diamond (2002, p. 495) conjectured that neuroimaging “tools” would enable the many unanswered questions surrounding the development of the PFC and the associated cognitive functions to be answered. Whilst methods such as electroencephalography (EEG)^1^ and functional Magnetic Resonance Imaging (fMRI) continue to provide many benefits to the developmental researcher, each comes with a set of limiting factors that may impose barriers to the investigation of the neural processes that underpin early cognitive development. Although not without its own limitations, substantial advances in functional Near-Infrared Spectroscopy (fNIRS) technology in more recent years have helped to significantly shape the study of the developing brain, allowing researchers to begin to unlock the neural correlates of early cognition.

The following provides a brief overview of these three commonly used neuroimaging techniques:

**EEG**

As a non-invasive method of measuring direct electrical activity in the brain (Michel & Murray, 2012), EEG is useful in uncovering the neural underpinnings of cognitive functions. This technique is frequently used in developmental research with infants and young children due to the relatively low level of participant tolerance needed (Lloyd-Fox, Blasi, & Elwell, 2010). EEG provides information about the size and frequency of a neuronal signal (power) and, due to its excellent temporal resolution of tens to hundreds of milliseconds (Luck, 2005), researchers are able to extract coherence data that provides information about functional connectivity between brain regions during cognitive tasks (e.g., Bell and Wolfe, 2007, Bell et al., 2007). However, the poor spatial resolution of this technology means it is difficult to discriminate the origin of neuronal signals, and so source localisation with EEG is not as accurate as with other techniques, such as fMRI (Burle et al., 2015). For more in-depth reviews on the use of EEG in developmental research, see, Bell and Cuevas (2012), and Saby and Marshall (2012).

**fMRI**

fMRI records the blood-oxygen-level-dependent (BOLD) response that occurs during neuronal firing (see, Barth & Poser, 2011), offering an indirect measurement of neuronal activity to researchers investigating the neural underpinnings of cognitive functioning. Whilst the temporal resolution of fMRI is limited by the relatively slow BOLD response, its excellent spatial resolution enables researchers to produce images of spatially localised brain activation (Dalenberg, Hoogeveen, & Lorist, 2018). fMRI is not frequently used in developmental research with very young participants due to its expensive cost, the constraints of the scanner environment, and the high level of tolerance it requires from participants (Lloyd-Fox et al., 2010). However, for a review of the ways in which fMRI can be effectively utilised in developmental cognitive neuroscience research, see Madhyastha et al. (2018).

**fNIRS**

fNIRS is a neuroimaging technique that is gaining popularity among neurodevelopmental researchers as it boasts major advantages for infant research (Lloyd-Fox et al., 2010, Wilcox and Biondi, 2016). Unlike fMRI, fNIRS does not require the participant to lie in a confined scanner environment, and it is more robust to movement (Aslin and Mehler, 2005, Cutini and Brigadoi, 2014). This method therefore overcomes many methodological limitations as now infants and toddlers are able to participate whilst awake, in a natural environment, and undertaking tasks that involve motor responses (see Fig. 1).

fNIRS involves placing a cap containing sources and detectors of near-infrared light on the participant’s head. The system then records changes in light absorption levels between sources and detectors (so-called ‘channels’). Since oxygenated and deoxygenated haemoglobin have different absorption spectra in the near-infrared range, it is possible to use fNIRS to measure the changes in concentration of oxygenated and deoxygenated haemoglobin in localised brain regions in response to a stimulus. Neuronal activity in a brain area is signalled by an increase in oxygenated haemoglobin and a decrease in deoxyhaemoglobin, compared to a baseline of relative rest (Lloyd-Fox et al., 2010). As fNIRS produces better spatial localisation than EEG, it allows for more accurate measurement of activity in brain structures associated with behaviours and functions of interest (Emberson, Zinszer, Raizada, & Aslin, 2017). However, because the near-infrared light cannot reach subcortical regions in children and adults, fNIRS technology is unable to provide information about the contribution of active subcortical areas that may play a role (Wilcox & Biondi, 2015). Nonetheless, fNIRS overcomes many of the drawbacks of other neuroimaging methodologies used with infants and young children.

Firstly, this review will explore the neural substrates of each of the three core EF components and how these change across infancy and throughout childhood. The neural substrates of each EF component will be discussed separately in light of Miyake and Friedman’s (Friedman and Miyake, 2017, Friedman et al., 2008, Miyake and Friedman, 2012, Miyake et al., 2000) model of executive function (see Box 2). Diamond, 2002, Friedman et al., 2008 observed that little research existed that identified the neural circuitry of early EF abilities, most likely due to the difficulties involved in testing infants and young children. In hindsight, another factor was probably also the lack of suitable neuroimaging techniques for this age group at the time.

Secondly, the current review will explore the structural, anatomical and biochemical development of the PFC, as well as the relationship between the protracted maturation of the PFC and the observed protracted developmental progression of EF abilities across childhood and into early adulthood. Whilst this relationship was emphasised and reviewed by Diamond (2002), relatively little was known at the time about the association between PFC maturation and the development of EF abilities. Recent research reviewed in this section will address new evidence for the association between PFC maturational changes and improvements in EF ability, as well as evidence for the existence of periods of rapid developmental change beyond the early childhood years.

Thirdly, Diamond (2002, p. 494) raised questions regarding the developmental timetable of PFC connectivity and its relationship with the age-related EF improvements evident in the literature at the time. For example, whilst the key role of the dorsolateral PFC (DL-PFC) in EF development was highlighted in Diamond’s chapter (e.g., Bell and Fox, 1992, Bell and Fox, 1997, Diamond, 1985, Diamond, 1988, Diamond, 1991, Fox and Bell, 1990), research existed which hinted that other brain regions, such as the anterior cingulate cortex (ACC), may play an important role in EF (Bush et al., 2000, Carter et al., 1999, Cohen et al., 2000, Posner and Rothbart, 1998). This led Diamond to highlight the potential role of connectivity within different prefrontal regions and between the PFC and other regions of the brain. Therefore, the third section of this review will focus on presenting recent evidence that PFC connectivity is key in EF development.

Finally, throughout this review we will survey a body of evidence from recent neuroimaging studies that provides a neuroscientific perspective on Diamond's (2002) unanswered questions. In the time that has passed since Diamond's (2002) review, the literature in this field has evolved and expanded, deepening our understanding of the neural substrates of EF and the developmental trajectories they follow. This expansion in knowledge is in part a result of technological advances that have allowed neuroimaging techniques such as functional magnetic resonance imaging (fMRI) and, more recently, functional near-infrared spectroscopy (fNIRS) to extend the developmental researcher’s toolkit (see Box 1). As a consequence, researchers have been able to measure neural activation that was previously difficult to measure, particularly in infancy and at pre-school age, or only had crude spatial localisation, such as in electroencephalogram (EEG) and event-related potential (ERP) studies. This, along with the increasing use of age-appropriate and well-validated behavioural tasks, has allowed a broadened understanding of EF during early development.

Box 2 **Models of executive function**

A separate debate that exists within the literature concerns the structure and organisation of EF. Since much of the research discussed in this review subscribes to the popular *unity-yet-diversity* model proposed by Miyake, Friedman and colleagues (Friedman and Miyake, 2017, Friedman et al., 2008, Miyake and Friedman, 2012, Miyake et al., 2000), it is important to discuss this and other models within the EF literature in order to provide a broader perspective on the evidence presented. In 2000, Miyake et al. posited that EF is a hierarchical construct consisting of a domain-general unitary entity and its three dissociable components (updating/WM, shifting and inhibition). These elements are thought to be mediated by a fronto-parietal network (Niendam et al., 2012).

More recent factor analytic work (Friedman and Miyake, 2017, Miyake and Friedman, 2012) has seen the introduction of a ‘common EF’ factor that represents the unity of the three core EF abilities. The diversity of EF exists in the factors that are specific to each component EF (e.g., updating-specific and shifting-specific factors). Interestingly, the factor analytic evidence suggested no specific factor for inhibition/inhibitory control. Therefore, in their most recent model, Miyake and Friedman (Friedman and Miyake, 2017, Miyake and Friedman, 2012) propose that inhibition is entirely subsumed under ‘common EF’. However, Miyake and Friedman’s conclusions are largely based on studies of young adults and therefore cannot be straightforwardly generalised to the structure of EF in childhood.

Although many studies of pre-school and school-aged children have adopted the *unity-yet-diversity* model (Asato et al., 2006, Huizinga et al., 2006, Lehto et al., 2003, St Clair-Thompson and Gathercole, 2006), a body of factor analytic and meta-analytic research has demonstrated that EF may occupy a more unified structure in the childhood period (Brydges et al., 2014, McKenna et al., 2017, Wiebe et al., 2008, Wiebe et al., 2011). For example, factor analytic work by Wiebe et al., 2008, Wiebe et al., 2011) found that performance of 2–6 year old children on a set of tasks thought to tap into different EF domains, in fact loaded onto a single common EF factor. To explain these results, Wiebe et al. (2008) turned to neuroimaging work by Duncan and colleagues (Duncan and Miller, 2002, Duncan and Owen, 2000). Duncan and colleagues proposed a unified model known as the ‘adaptive neural coding framework’ to explain the structure of EF, claiming that EF is a unitary, domain-general construct. This function recruits the same specialised frontal pathway (mid-dorsolateral PFC, mid-ventrolateral PFC and the ACC) in different ways depending on task demands for a range of challenging cognitive tasks. This model provides an alternative view to the *unity-yet-diversity* theory of Miyake and colleagues (Friedman and Miyake, 2017, Friedman et al., 2008, Miyake and Friedman, 2012, Miyake et al., 2000), and recent single cell and fMRI research from Duncan (2010) does indeed confirm a common pattern of frontal and parietal activity that is associated with both fluid intelligence and a range of diverse cognitive demands. However**,** Friedman and Miyake (2017) have since argued that this evidence simply supports the unity construct within their *unity-yet-diversity* model, so the debate continues.

McKenna et al. (2017) have recently proposed that a systematic *developmental* model of EF would better encompass the changes observed in the structure of EF, and the underlying neural correlates during childhood and adolescence. In their meta-analytic study of fMRI data, they identified separable areas of neural activation for shifting and updating in young adolescents (13–18 years), but were unable to find evidence of separable components in children (6–12 years) (McKenna et al., 2017). As in the work by Miyake and Friedman (Friedman and Miyake, 2017, Miyake and Friedman, 2012), this meta-analysis found no evidence for an inhibition-specific factor that was separable from the common neural activation in either age group (McKenna et al., 2017). Taken together, the evidence from McKenna et al.'s (2017) meta-analysis demonstrates the existence of common neural activation across all EF tasks in both age groups, providing support for Miyake and Friedman’s (Friedman and Miyake, 2017, Miyake and Friedman, 2012, Miyake et al., 2000) concept of ‘unity’. Importantly however, McKenna et al.'s (2017) meta-analysis also provides evidence that the structure of EF changes across development, from one that is largely unified and recruits a common neural network in early-to-middle childhood, to one that involves more diverse components that each recruit specific neural networks. This is largely in line with evidence that a shift from globalised neural activation to refined, localised activation during EF tasks occurs in the PFC across childhood (see the section “From global to local brain activation: a refinement and specialisation process”).

## The development and neural substrates of EF components

Almost two decades ago, Diamond's (2002) chapter on the normal development of the PFC provided a substantial review of evidence regarding the anatomy and biochemistry of the PFC that underpinned normal cognitive functioning. However, key questions about the development and neural substrates of EF remained unanswered: to what extent is the PFC involved in different cognitive functions and their associated behavioural tasks? What other brain regions are involved? What are the different developmental profiles of the neural system that underlies each cognitive function? (Diamond, 2002, p. 493).

At the time, the field of developmental psychology was on the cusp of a boom in neuroimaging research that would uncover answers to some of these questions. Indeed, since then, numerous research studies that have made use of the vast advances in neuroimaging technology have been published. These studies have confirmed that different regions within the PFC are specialised to sub-serve different components of EF, whilst also forming part of a wider network of brain regions that function to facilitate EF across different tasks (see the section “Frontal connectivity: from diversity to specialisation”). In the current section, we will highlight research investigating the neural substrates of each of the core EF components. (For discussion of commonality across EF domains and the associated brain networks, see Box 2 and Box 4).

### Working memory

According to Baddeley (1992), working memory (WM) refers to a limited capacity cognitive system that enables the temporary storage and manipulation of information. Following a review of evidence at the time, Diamond (2002) identified the DL-PFC as a key player in WM development. However, whilst confirming the importance of the DL-PFC, much of the more recent research has also demonstrated the important roles of regions beyond the DL-PFC, as well as the significance of connections between these areas, in WM functioning. It is important to note that, whilst this review will follow Diamond's (2002) terminology of ‘DL-PFC’, more recent EEG and fNIRS studies tend to refer to this region more broadly as the 'lateral PFC'. This terminology is likely used to account for the limited spatial resolution of these techniques, which makes it difficult to localise a more specific active region of the PFC (see Box 1).

In her 2002 chapter, Diamond’s summary of her previous research (Diamond, 1988, Diamond, 1991) highlights the relationship between the A-not-B task (a measure of WM and IC, see Box 3) and the DL-PFC. This research identified that human infants (7½ − 9 months), infant monkeys (1½ − 2½ months), adult monkeys with removed DL-PFC and adult monkeys injected with MPTP (which reduces dopamine in the PFC) all fail the A-not-B task. Similarly, neuroimaging evidence from EEG studies with human infants suggested that age-related improvements on the A-not-B task were linked to changes in patterns of electrical activity over the frontal and parietal cortices (Bell and Fox, 1992, Bell and Fox, 1997, Fox and Bell, 1990). This evidence provided strong support for a link between performance on the A-not-B task and the DL-PFC. In fact, it was claimed by Diamond (2002) to be the strongest brain-behaviour relationship in all of cognitive neuroscience at the time.

Box 3 **The A-not-B/Delayed Response Task**

Early work by Jacobsen (1935) first introduced the delayed response task as a means of investigating the functionality of the DL-PFC in monkeys. A version referred to as the A-not-B task was also developed by Piaget (1954) for use with infants. Since then, the A-not-B/delayed response task has been frequently used with infants and young children to investigate the function of the PFC. The A-not-B task involves an object (usually a toy) being hidden in view of the infant under location A. After a brief delay, the infant is allowed to search for the object (see Fig. 2). Once the infant has successfully found the object at location A multiple times, the object is hidden under location B. Infants are considered to make the A-not-B error (or perseveration error) if they continue to look under location A, despite having just seen the object being hidden under location B. Whilst in the A-not-B task the length of the delay being imposed is determined by the infant’s performance, the delayed response task follows a fixed delay sequence that does not depend on the infant’s performance. According to Diamond (2002), both tasks tap into EF ability because they require aspects of working memory as well as inhibition of a prepotent response. Today, the A-not-B task is still widely used as a tool to provide evidence for infants’ increasing EF task performance during the latter half of the first year of life (Bell and Wolfe, 2007, Cuevas et al., 2012, Holmboe et al., 2008, Holmboe, Bonneville-Roussy, et al., 2018, Nelson et al., 2008). Since the A-not-B and delayed response tasks are very similar, we use ‘A-not-B’ to refer to this type of task in the current review.

More recently, researchers have been able to investigate the rudimentary beginnings of WM in infancy using fNIRS, an increasingly popular neuroimaging technique among developmental researchers (see Box 1). In a seminal fNIRS study, Baird et al. (2002) found that 5–12 month old infants recruit the DL-PFC when holding objects in visual WM, confirming the direct role of this area of the PFC in early WM development. However, a more recent fNIRS study found that regions beyond the DL-PFC also play an important role in WM functioning during infancy; Wilcox and Biondi (2016) revealed that, during a WM maintenance task, infants showed decreasing task-related activation in the posterior temporal cortex from 3–12 months, and yet activity in the occipital cortex remained unchanged throughout this age range. The authors proposed that this decrease in activation reflects the functional specialisation of visual WM over the latter half of the first year of life. EEG research has also elucidated WM development during infancy. Research by Bell (2012) found that successful A-not-B performance was related to increased fronto-parietal activation at 8 months. Similarly, Cuevas et al. (2012) found that, whilst at 5 months an increase in EEG coherence was present across the entire scalp during A-not-B performance (relative to baseline), at 10 months this coherence was limited to the medial frontal and occipital electrode sites, suggesting at least some degree of specialisation during the first year. Not only do these recent studies highlight the involvement of brain regions beyond the DL-PFC in early WM development, they also shed light on Diamond's (2002) notion that the PFC does not work in isolation (p. 494); a more detailed discussion of these findings from a specialisation perspective will follow in a later section (“From global to local brain activation: a refinement and specialisation process”).

Beyond infancy, there is evidence that the lateral PFC continues to play an important role. In a recent fNIRS study of 3–7 year olds, Perlman, Huppert, and Luna (2016) found increased activation of the lateral PFC during a WM task compared to rest. This study found that, as expected, accuracy and response speed during a WM task strengthened with age. At the neural level, this improvement was mirrored by increased DL-PFC activation in older children. Furthermore, when the delay before responding was increased, activation in the lateral PFC also increased. These results suggest that WM develops incrementally during early childhood and that the lateral PFC is involved throughout, showing activation that is sensitive to WM load. However, the parietal cortex has also been extensively implicated in childhood WM development. Similar fNIRS research by Buss, Fox, Boas, and Spencer (2014) investigated the visual WM capacity of 3–4 year old children during a change-detection task. Results indicated that during periods of WM maintenance, children displayed increased activation in both the frontal and parietal cortex. The increase in activation in the parietal cortex was higher in 4 year olds than in 3 year olds, perhaps reflecting the sensitivity of this region to visual WM load (Buss, Fox, Boas, & Spencer, 2014).

Further evidence for the involvement of less traditional WM areas during development comes from a longitudinal study by Ullman, Almeida, and Klingberg (2014), who used structural and functional MRI to track changes in the DL-PFC and subcortical structures. It was found that activation of the DL-PFC in 6 year old children was indicative of present WM ability. However, DL-PFC activation was not predictive of future WM ability. Instead, future WM ability was predicted by the structure and activation of the basal ganglia and thalamus. An fMRI study by Crone, Wendelken, Donohue, van Leijenhorst, and Bunge (2006) indicated that, whilst children and adults equally activated the ventrolateral PFC during WM tasks, children failed to recruit the right DL-PFC and bilateral superior parietal cortex. Finally, an fNIRS study of 4–10 year old children by Smith et al. (2017) indicated that the right inferior frontal gyrus and the orbitofrontal cortex may be involved in non-spatial WM. However, this study is among the first to employ fNIRS with children using a non-spatial WM task, and therefore replication is needed to further clarify the localisation of brain activation when the content to be retained is non-spatial in nature (Smith et al., 2017).

In summary, the frontal and parietal cortices have been identified as core neural substrates of WM from infancy onwards, and subcortical structures have also been shown to be involved (Alcauter et al., 2014, Ullman et al., 2014). However, it is important to note that the connectivity between frontal and parietal cortices, and the network they form, are also important in facilitating the development of WM. This will be discussed in detail in a later section (see, “Frontal connectivity: from diversity to specialisation”).

Box 4 **Can working memory and attention be distinguished from one another?**

The argument as to whether the two cognitive constructs of WM and attention can really be distinguished from one another was raised by Diamond in 2002. In her chapter, Diamond posited that the difference between the two may only be semantic (p. 492), arguing that evidence pointed to the same underlying PFC system facilitating both selective attention and working memory (e.g., Awh & Jonides, 2001). This topic has since been the subject of much research and theoretical discussion in the field.

Although not included in Miyake et al.’s (Miyake and Friedman, 2012, Miyake et al., 2000) model of EF, some researchers have put forward the idea that attention may be a necessary first-step towards exhibiting EF skills (Garon et al., 2008, Hendry et al., 2016, Holmboe, Bonneville-Roussy, et al., 2018). In fact, many describe attentional control as a core component of WM (Amso and Scerif, 2015, Astle and Scerif, 2011, Baddeley, 1996, Cowan and Morey, 2006, Kane and Engle, 2002, Klingberg et al., 2002). A major model of attention proposed by Posner and Rothbart (Posner and Rothbart, 2007, Rothbart and Posner, 2006, Rothbart et al., 2007) describes an *executive attention network* as having an important association with EF (Rothbart & Posner, 2001). Using fMRI, Posner, Rothbart, Sheese, and Voelker (2012) were able to study neonates to provide an insight into the very earliest stages of the development of attention. Even in newborn infants, parietal areas displayed strong connectivity to lateral/medial frontal areas; areas that have been previously implicated in both the executive attention network (Posner and Rothbart, 2007, Rothbart and Posner, 2006, Rothbart et al., 2007) and EF (Gao et al., 2009, Rothbart and Posner, 2006, Rothbart et al., 2007). This executive network has been found to include the anterior cingulate cortex, the lateral ventral PFC and the basal ganglia, and is modulated by dopamine (Posner & Fan, 2008). Interestingly, these areas overlap substantially with the regions found to be active during WM tasks (Crone et al., 2006, Nagy et al., 2004, Scherf et al., 2006, Sowell et al., 2003, Sowell et al., 2004).

Certainly, there appears to exist an overlap in the developmental trajectories and neural underpinnings of elements of both executive attention and WM. Cuevas and Bell (2014) found evidence that infant attention at 5 months is associated with early childhood EF (at 24, 36 and 48 months). The authors discussed this as evidence of Posner & Rothbart’s (Posner and Rothbart, 2007, Rothbart and Posner, 2006, Rothbart et al., 2007) executive attention network, but with a somewhat earlier emergence than previously suggested. Within infancy, findings have been contradictory, with some research indicating that attention does not form the foundation for EF abilities at the end of the first year (Holmboe, Bonneville-Roussy, et al., 2018) and other research indicating that it does (Blankenship et al., 2019). It seems likely that the type of attention assessed is important here – the study by Holmboe, Bonneville-Roussy, et al. (2018) used primarily measures of saccadic reaction time (see, Geeraerts et al., 2019, for a similar negative finding with a saccadic reaction time measure in older infants), whereas Blankenship et al. (2019) focused on peak look duration and shift rate to and from a video. Another recent study by Cheng, Kaldy, and Blaser (2019) found that focused attention, as assessed by pupil response during stimulus encoding, directly predicted subsequent WM performance in 13 month olds. Finally, research in toddlers by Veer, Luyten, Mulder, van Tuijl, and Sleegers (2017) indicated that selective attention at 2½ years was predictive of WM and response inhibition at 3 years. Overall, the evidence suggests important links between the development of at least some aspects of attention and WM abilities during the first few years of life, potentially leading to a convergence of processes that allow for the emergence of higher-order EF skills during the pre-school period.

### Inhibitory control

Inhibitory control refers to the process of preventing (inhibiting) an automatic or prepotent response in order to achieve a behavioural or cognitive goal. The behavioural developmental trajectory of IC begins towards the end of the first year of life (Diamond, 2002, Diamond et al., 2007, Garon et al., 2008, Wolfe and Bell, 2007) with research suggesting that some early forms of inhibitory (Holmboe, Bonneville-Roussy, et al., 2018) and attentional (Courage, Reynolds, & Richards, 2006) control emerge already at around 6 months of age. Following this, IC shows a prominent and rapid improvement during the toddler and preschool years (Friedman et al., 2011, Garon et al., 2008, Garon et al., 2014, Holmboe, Larkman, et al., 2018, Simpson and Riggs, 2005), increasing at a more steady pace throughout middle childhood (Best and Miller, 2010, Best et al., 2009), before eventually reaching adult levels in early adolescence (van den Wildenberg and van der Molen, 2004, Williams et al., 1999).

Adding a new dimension to the behavioural literature existing at the time, Diamond (2002) reviewed neuroimaging studies that implicated the dorsal and ventro-lateral PFC in IC tasks, such as the Go/No-Go task (Casey et al., 1997, Konishi et al., 1998 T. Tsujimoto et al., 1997), findings that have been replicated in several later fMRI studies (Bunge et al., 2002, Durston et al., 2002, Durston et al., 2006, Smith et al., 2017, Tamm et al., 2002; see also the section on “From global to local brain activation: a refinement and specialisation process”). The DL-PFC in particular, has been implicated when the task demands require elements of both WM and response inhibition, and some evidence indicates this is more so in infancy and early childhood when these two EF components are still co-dependent (Casey et al., 1997, Diamond, 2002, Tamm et al., 2002). Activation in the DL-PFC during response inhibition tasks has been found to reduce with development (Durston et al., 2006, Tamm et al., 2002). Parietal activation (and changes in this activation with development) has also been observed in many recent neuroimaging studies of IC, as has the involvement of the striatum (Bunge et al., 2002, Durston et al., 2006, Durston et al., 2002, Mehnert et al., 2013).

The advent of fNIRS (see Box 1) now allows an investigation of the neural correlates of IC at an earlier age than most fMRI studies. Recently, Mehnert et al. (2013) employed fNIRS to measure the neural substrates of IC in children aged 4–6 years compared to adult participants, using the Go/No-Go task. It was found that, whilst adults activated the right frontal and parietal regions during No-Go (inhibition) trials, children maintained a high level of right frontal and parietal activation in both Go and No-Go trials, perhaps indicating the high inhibitory demand of the task in young children (note, however, that a further substantial increase in activation was seen during No-Go trials in the children). Another recent fNIRS study by Moriguchi and Shinohara (2019) used a more emotionally charged inhibitory control task, the ‘Less Is More’ (LIM) task, with 3–4 year olds. Stronger right inferior frontal cortex (rIFC) activation was found when children were able to inhibit pointing to the larger reward and instead point to the smaller reward (the counter-intuitive action needed to receive the larger reward). The finding of rIFC activation during an IC task with a ‘hot EF’ aspect is interesting, as it suggests that young children may engage the same neural substrates in reward-driven contexts as in the much more heavily investigated (at least in terms of neuroimaging research) ‘cool EF’ contexts (for a review of hot and cool aspects of EF, see, Zelazo & Carlson, 2012).

Whilst the rIFC (sometimes also referred to as the right ventrolateral PFC) has long been known to play a key role in the inhibition of prepotent responses, particularly in the Stop-signal task (Verbruggen & Logan, 2008), the exact neural network involved was unknown to Diamond in 2002. Aron et al., 2004, Aron et al., 2014 have since proposed that the process of stopping (i.e., inhibiting) a motor response occurs when the rIFC sends a signal to the subthalamic nucleus of the basal ganglia, which then supresses thalamocortical output and thus prevents the occurrence of a motor response. Aron et al., 2004, Aron et al., 2014 proposal that the rIFC’s main role is stopping prepotent responses is not undisputed (see, Banich & Depue, 2015). Nevertheless, developmental neuroimaging research has confirmed the involvement of this brain area in IC. Many studies have linked the fronto-basal-ganglia network, which includes the rIFC, to performance on IC tasks (Aron et al., 2007, Durston et al., 2002, Mehnert et al., 2013, Moriguchi and Shinohara, 2019, Rubia et al., 2007, Smith et al., 2017). It is worth noting, however, that there is some evidence that the specialisation of the rIFC as the *key substrate* of IC occurs relatively late in development, with the transition happening during late childhood and adolescence (Durston et al., 2006, Rubia et al., 2007, Schroeter et al., 2004). Furthermore, on occasion, results have been contradictory, with developmental changes in *left* or *bilateral* IFC activation being observed (Durston et al., 2002, Rubia et al., 2007, Tamm et al., 2002). For example, Tamm et al. (2002) found developmental increases in activation in the left IFC rather than the right IFC, and, using fNIRS during a ‘hot’ IC task (the Choice Delay of Gratification Task), Moriguchi, Shinohara, and Yanaoka (2018) found contradictory results to the adult literature in that children activated the rIFC more when they *failed* to delay gratification. A meta-analysis or large-scale replication of these neuroimaging studies would therefore be useful in the future, in order to clarify the exact ways that the neural substrates of IC change with development.

### Cognitive flexibility

The neural system that underpins the EF ability of cognitive flexibility, or set shifting (the ability to flexibly switch between cognitive tasks or concepts), has been widely investigated since Diamond's (2002) chapter. Whilst it is included as a core component in their model of EF (Miyake and Friedman, 2012, Miyake et al., 2000), Miyake et al. (2000) claim that shifting may also involve inhibition, in the form of reducing interference, as well as some elements of WM.

#### Set shifting

A body of work by Brass and colleagues (Brass et al., 2003, Brass et al., 2005, Derrfuss et al., 2005) has indicated the important contribution of frontal regions, including the lateral PFC and the inferior frontal junction (IFJ) to performance on set shifting tasks in adults. From a developmental perspective, fMRI research from Morton, Bosma, and Ansari (2009) indicated that children and adults recruit different regions during a rule-switching task. Whereas only children activated an area around the right superior frontal sulcus during switching, adults (but not children) showed additional activation in the left superior parietal cortex and the thalamus. This evidence demonstrates the developmental changes in frontal and parietal regions that enable adult-like cognitive flexibility.

In recent years, the neural correlates of set shifting have also been investigated in pre-school children. Moriguchi and Hiraki, 2009, Moriguchi and Hiraki, 2011 used fNIRS to measure the concentration of oxygenated haemoglobin in the ventrolateral PFC, an area often implicated in EF, particularly inhibitory control and task-switching (Aron et al., 2004, Aron et al., 2014, Durston et al., 2006; see also the section “Inhibitory control”). Moriguchi and Hiraki (2009) found that 3 year olds who were able to successfully switch between rules in the dimensional change card sort task (DCCS) showed a bilateral increase in oxygenated haemoglobin in the ventral and dorsolateral PFC, whereas those who were unable to flexibly shift task did not. In a subsequent longitudinal follow-up study (Moriguchi & Hiraki, 2011), it was found that both behavioural performance and PFC activation increased between 3 and 4 years. Furthermore, children who performed well on the task at age 3 showed initial right IFC activation changing to significant bilateral IFC activation a year later. On the other hand, poor performers on the DCCS at age 3 had no initial IFC response and only showed an increase in left IFC activation by age 4. This research provides evidence for the neural underpinnings that vitally support the improved EF seen in young pre-school aged children, as well as highlights the substantial involvement of the ventral PFC in successful set shifting, even early in development.

Finally, a recent well-powered fNIRS study in 4-5 year old children by Quiñones-Camacho, Fishburn, Camacho, Wakschlag and Perlman (2019) found an increase in neural activation in the left DL-PFC during a cognitive flexibility task (the ‘Pet Store Stroop task’). Interestingly, children who had strong attentional control skills (as rated by parents) performed better on the task and also exhibited lower task-related activation in the DL-PFC. This could indicate that these children had more efficient neural processing in the DL-PFC or a more integrated brain network mediating cognitive flexibility (see the section “From global to local brain activation: A refinement and specialisation process”).

#### Conflict resolution

Some researchers (Botvinick et al., 2001, Fan et al., 2003, Gerardi-Caulton, 2000, Rothbart et al., 2003) have pointed out that both set shifting and conflict resolution tasks often recruit the anterior cingulate cortex (ACC). Indeed, it has been proposed that the ACC plays an evaluative role in monitoring the environment for cognitive conflict, and when conflict is found, the ACC, via its connectivity with the PFC, strengthens its attention-guiding rules and thus allows for cognitive flexibility (Botvinick et al., 2001, Braver et al., 2007). When these rules are strengthened, processing becomes task-relevant and conflict effects on subsequent incongruent trials, in tasks such as the DCCS, are reduced (Waxer & Morton, 2011).

However, it is only with functional maturity that the ACC facilitates successful set shifting. Morton and Munakata (2009) reviewed evidence that infants, compared to adults, are prone to dysfunctional cognitive control in object search (Munakata, 1998), whilst children (aged 5–9 years) make more perseveration errors on flexible rule use tasks (Chevalier and Blaye, 2009, Morton and Munakata, 2002). Morton and Munakata (2009) proposed that children have difficulty maintaining strong active representations of the attention-guiding rules that are facilitated by ACC-PFC connectivity (for more detail about connectivity between brain regions, see the section on “Frontal connectivity: from diversity to specialisation”). Further evidence for the under-developed functional connection between the ACC and the PFC in children comes from a study by Waxer and Morton (2011), where results indicated that whilst adults and adolescents recruited the ACC to respond to conflict during DCCS tasks, children did not. The findings from this study suggest that whereas adults and adolescents utilise prior conflict to prepare for the future, children respond to cognitive challenges as they occur.

## The development of the PFC

It has long been known that the PFC plays a pivotal role in EF across the lifespan, with many research studies reviewed by Diamond (2002) finding evidence of its active involvement during the performance of EF tasks (Bell and Fox, 1992, Bell and Fox, 1997, Fox and Bell, 1990, Roberts et al., 1998). Even with methodological advances allowing much more detailed mapping of cognitive function to neural substrates in the years since Diamond's (2002) review, research continues to identify the involvement of the PFC in EF throughout development (e.g., Aron et al., 2004, Aron et al., 2014, Baird et al., 2002, Brass et al., 2003, Brass et al., 2005, Perlman et al., 2016, Moriguchi and Hiraki, 2011). However, at the time of writing, Diamond (2002) stated that relatively little was known about the developmental changes in the prefrontal neural system that underpins EF. As a result, questions were raised regarding the relationship between the structural and functional maturation of the PFC and the cognitive advances seen in EF task performance. We turn to this topic next.

### Structural PFC development

Taking up almost a third of the cerebral cortex, the PFC is one of the last brain regions to fully mature; it takes over two decades to reach full structural and functional maturity (Casey et al., 2005, Diamond, 2002, Huttenlocher, 1979, Huttenlocher, 1990, Kostović et al., 1988, Sowell et al., 1999). Researchers have observed that structural PFC maturation consists of both progressive (myelination, neuron proliferation, synaptogenesis) and regressive (cell death, synaptic pruning, loss in grey matter) changes (Casey et al., 2006, Gogtay et al., 2004, O’Hare et al., 2008). Interestingly, the physical maturation of the frontal lobe appears to parallel the advances seen in cognitive abilities throughout childhood and adolescence (Bathelt et al., 2017, Bathelt et al., 2018, Bathelt et al., 2018, Gibson, 1991, Gogtay et al., 2004, Goldman-Rakic, 1987, Huttenlocher, 1994, Romine and Reynolds, 2005).

Increases in cortical volume, synapses and dendritic trees during the PFC maturation period serve to facilitate information processing by forming connections between the PFC and other cortical areas. Research demonstrates that an increase in dendritic trees in layer III of the PFC occurs between 6 and 12 months (Koenderink, Uylings, & Mrzljak, 1994), and a peak in synaptogenesis in the middle frontal gyrus occurs between 12 and 18 months of age (Huttenlocher & Dabholkar, 1997). Similarly, more recent post-mortem research notes a process of synaptogenesis in the PFC within the first decade of life before a reduction in synapses occurs throughout adolescence and into adulthood (Glantz, Gilmore, Hamer, Lieberman, & Jarskog, 2007).

However, synaptogenesis is not the only change in the PFC that follows a protracted time course; studies of glucose metabolism provide evidence of a similar prolonged maturation period. Early positron emission tomography (PET) studies found that glucose metabolism in the PFC began to increase at around 6–8 months and continued to increase until 3–4 years, whereas in the somatosensory cortex glucose utilisation was high from birth (Chugani and Phelps, 1986, Chugani et al., 1987). Furthermore, research by Franceschini et al. (2007) utilised frequency-domain near-infrared spectroscopy (see Box 1) to measure blood volume and oxygen consumption in healthy infants across the first year of life. It was found that increases in cerebral blood volume across the cortex were closely linked to increases in the metabolic demand of neurons. Specifically, Franceschini et al. (2007) found that cerebral blood volume in the frontal region increased linearly from birth before reaching its plateau at approximately 8–9 months of age, whereas an exponential increase was seen in occipital, parietal and temporal regions only within the first 2½ months of life. Together these findings suggest that a parallel time course of prolonged maturation exists between the structural and metabolic changes in the PFC that occur during the first 2–4 years of life.

Region-specific changes with development also occur in the spread of myelin across the cortex. Myelination is crucial for normative cognitive development, as it allows for efficient and synchronised communication between neural systems (Deoni et al., 2011, Miller et al., 2012). Important early post-mortem work by Yakovlev and Lecours (1967) observed that myelination in the infant cortex follows a hierarchical pattern, with subcortical brain regions achieving myelination before brain regions involved in higher-order functions, such as the PFC. Later histological work by Kinney, Brody, Kloman, and Gilles (1988) confirmed this by demonstrating that myelination in subcortical regions occurs much earlier (e.g., central tegmental tract reached mature myelination at 9 weeks of age) than myelination in higher cortical areas, such as the frontal pole, which reaches full myelination at 24 weeks (6 months) of age. More recently, Deoni et al. (2011) used a new MRI technique to map the spread of myelination *in vivo* across the cortex during the first year of life. The cerebellum, pons and internal capsule were among the first to myelinate, with myelination reaching occipital and parietal lobes around 4–6 months, and the frontal and temporal lobes last, at around 6–8 months. This again provides evidence for the relatively late maturation of the frontal lobe in comparison to other brain regions.

Finally, recent histological studies indicate that the human frontal cortex is also the target of substantial migration of immature neurons in the first few postnatal months; this is interesting, as most neural migration is completed during prenatal development so, once again, the frontal cortex appears to develop to a different schedule than most other cortical regions. Sanai et al. (2011) found that, not only did young neurons migrate into the olfactory bulb after birth, but a separate stream, the medial migratory stream (MMS), was also involved in migration into the ventro-medial PFC up until 4 months of age (but not beyond 8 months of age). Subsequently, Paredes et al. (2016) discovered much more extensive neuronal migration from what they termed the ‘Arc’, a dense layer of young neurons adjacent to the anterior body of the lateral ventricle. Many of these neurons reached dispersed regions of the PFC in the early postnatal period (ending around 3 months of age), where they developed characteristics of inhibitory interneurons. Although the functional consequences of this late neuronal migration into the human PFC is as yet unknown, Paredes et al. (2016) speculated that the inhibitory characteristics of these new interneurons could play an important role in the early plasticity and maturation of the frontal cortex.

Despite these changes in synaptogenesis, metabolism, myelination and neuronal migration in the PFC during the infancy and early childhood period, continued, gradual change occurs well beyond this age. Numerous structural MRI studies have shown that cortical thickness and volume develop following an inverted U-shaped trajectory, increasing initially in childhood and then declining during early adulthood (Gogtay et al., 2004, Shaw et al., 2008, Tamnes et al., 2013). Notably, this decline does not occur in the PFC until late adolescence; in contrast to most other cortical areas, which show a reduction in grey matter earlier in the teenage years (Gogtay et al., 2004). These findings mirror the histological work on synaptogenesis in the PFC by Glantz et al. (2007, see above). The inverted U-shaped trajectory has also been observed in the expansion in PFC volume across development. Research by Kanemura, Aihara, Aoki, Araki, and Nakazawa (2003) used structural MRI to measure PFC volume in individuals from infancy to adolescence. Results indicated that the PFC expands steadily in volume between 5 months and 8 years of age, then expands rapidly from 8–14 years, before declining in early adulthood.

### Behavioural correlates of structural PFC development

The evidence reviewed in the previous section clearly demonstrates the unique and protracted development of the PFC and the significant structural changes that occur during its process of maturation. In her 2002 review, Diamond prompted future researchers to identify the developmental changes in the PFC that underpin the cognitive performance improvements seen across childhood (p.494). As it happens, in the decades since then, in part due to the wider availability of neuroimaging technology, researchers have begun to elucidate the behavioural correlates of structural developments in the PFC.

Research on *direct* links between structural PFC development and cognitive performance in infancy and early childhood is still limited. However, there exists some evidence to suggest that the structural integrity of the corpus callosum and white matter tracts that connect the PFC to other brain regions during infancy are predictive of EF in later childhood (Ghassabian et al., 2013, Woodward et al., 2011). Early research by Koenderink et al. (1994) demonstrated a period of dendritic growth in the DL-PFC from 7½–12 months of age, coinciding with the period of rapid improvement on the A-not-B and object retrieval tasks (Diamond, 1985, Diamond and Doar, 1989). As a result, the latter part of the first year of life has been identified as a period of substantial EF development (Diamond, 2002). However, recent research suggests that some early forms of EF (in this case, IC) may emerge even earlier. Longitudinal research by Holmboe, Bonneville-Roussy, et al. (2018) found stability of individual differences in IC ability already from 6 months of age. Interestingly, this behavioural finding is consistent with a finding from Deoni et al.'s (2011) structural MRI research, which demonstrated frontal lobe myelination occurring from 6–8 months of age. Indeed, Deoni et al. (2011) suggested that myelination allows for the rapid and synchronised information processing that is required for many cognitive functions, including aspects of EF. Whilst an intriguing suggestion, it is important to note that in order to establish a direct link between structural PFC maturation and early EF performance improvements, it would be necessary to conduct research that both obtains structural MRI recordings and behavioural data from the same cohort of infants.

**Fig. 3:**
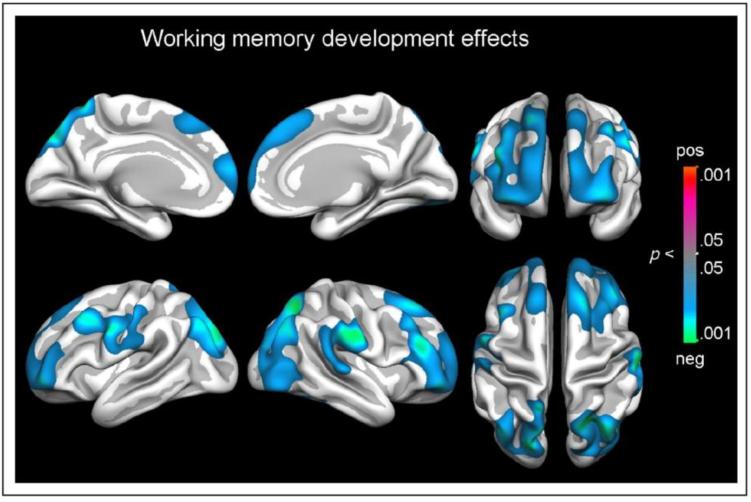
Figure taken from Tamnes et al. (2013), as published in the *Journal of Cognitive Neuroscience, 25 (10)*, and reprinted with permission from the publisher (*the Massachusetts Institute of Technology Press Journals*). The figure demonstrates that three clusters of cortical volume reduction were associated with improvement in WM performance in a longitudinal sample of children and adolescents aged 8–22 years. Longitudinal change in cortical and subcortical volumes was quantified by the use of Quantitative Anatomical Regional Change.

Despite early childhood (2–6 years of age) being a period of notable development in both EF task performance and structural brain changes (Best et al., 2011, Bolton and Hattie, 2017; see also section on “Structural PFC development”), studies investigating the behavioural correlates of structural brain development in this age group are generally lacking in the literature. This is likely due to the infeasibility of utilising structural MRI techniques with young children and the lack of age-appropriate tasks for measuring EF at the younger end of this age range (Anderson and Reidy, 2012, Holmboe, Larkman, et al., 2018). However, research with older children and adolescents has demonstrated that performance improvements on EF tasks *indirectly* parallel the structural changes in grey matter (Sowell et al., 2003, Sowell et al., 2004) and are *directly* associated with the structural changes in white matter (Nagy et al., 2004) that occur in the same fronto-parietal cortices that are recruited *during* EF task performance (see the sections “Frontal connectivity: from diversity to specialisation” and “The development and neural substrates of EF components”). In this age group, direct links between structural brain development and EF performance have also been demonstrated. For example, Tamnes et al. (2010) used structural MRI to collect measures of cortical thickness from participants aged 8–19 years, who also completed a set of EF tasks. Results indicated that, independent of age, performance on cognitive flexibility tasks was associated with thinner cortex clusters in parietal and frontal regions, including the left inferior frontal gyrus, whereas performance on the inhibitory control tasks corresponded with thinner cortex in occipital and parietal regions (but not frontal regions). In a continuation of this research, Tamnes et al. (2013) found that improvement in working memory at a 2-year longitudinal follow-up assessment was related to cortical volume reduction in the lateral PFC and in the posterior parietal cortex (see Fig. 3). Tamnes et al. (2013) argued that this demonstrates that structural maturation of fronto-parietal regions is directly related to the development of working memory. The authors also suggested that the processes of myelination and synaptic pruning in adolescence (which results in cortical volume changes) contribute to increased specialisation and processing efficiency in the fronto-parietal network, potentially underlying the continued development and refinement of executive function performance during late childhood and adolescence.

### The role of prefrontal dopamine during EF development

Diamond (2002) recognised the importance of neurotransmitters such as dopamine, acetylcholine, serotonin and noradrenaline in the PFC, yet their roles and resulting impact on executive functions remained unclear. Indeed, Diamond (2002) finished her chapter by posing several questions regarding the role of neurotransmitters (including dopamine) in the development of cognitive functions sub-served by the PFC, commenting that, at the time, very little evidence existed that could shed light on this question (p. 494). Nevertheless, the important role of prefrontal dopamine on EF performance had already been demonstrated by Diamond and colleagues’ ground-breaking research on children with the rare metabolic disorder phenylketonuria (PKU; Diamond, 2001, Diamond et al., 1997). Children with PKU are unable to metabolise phenylalanine, which can lead to lower levels of dopamine specifically in the PFC, especially if the condition is not carefully managed. As predicted by Diamond et al. (1997), the lack of prefrontal dopamine in children with PKU resulted in a selective deficit in tasks involving WM and inhibition.

Research conducted since Diamond’s (2002) review has begun to investigate the effect of genetic polymorphisms that affect dopamine regulation in the PFC. Since genetic methods are non-invasive, they are useful to developmental researchers as a way of identifying the relationship between prefrontal dopamine level and cognitive abilities. In particular, polymorphisms of the catechol-*O-*methyltransferase (COMT) gene are often studied, as the gene regulates an enzyme that degrades 60% of dopamine in the PFC (Karoum, Chrapusta, & Egan, 1994). A study by Tunbridge, Harrison, and Weinberger (2006) provided evidence that different COMT Val^158^Met genotypes result in different levels of dopamine in the PFC. Whilst Met homozygotes have the highest level of dopamine available in the PFC (since the breakdown of dopamine is less efficient), Val homozygotes have the lowest level of dopamine available. Heterozygotes have an intermediate level. In addition, several studies and meta-analyses have found significant associations between COMT genotype and prefrontal activation (Chen et al., 2004, Durstewitz and Seamans, 2008, Mier et al., 2010, Schacht, 2016, Slifstein et al., 2008).

Consistent with these findings, some studies have found that the Met-allele is associated with better performance on EF tasks (Diamond et al., 2004, Egan et al., 2001, Malhotra et al., 2002). Examining this in infancy, Holmboe et al. (2010) found that 9 month old infants with the Met/Met genotype showed greater sustained attention and were less distractible in the Freeze-Frame task (a measure of IC suitable for infants). Similar research by Markant, Cicchetti, Hetzel, and Thomas (2014) with 7 month old infants indicated that, whilst Val-carriers were more greatly influenced by novelty (and thus more distractible), Met-carriers had higher levels of sustained attention and behavioural regulation; results which support the findings of Holmboe et al. (2010). These studies highlight that a key variant of the COMT gene contributes significantly to individual differences in infant attention, behavioural regulation, and early EF. Furthermore, because of the substantial evidence that the COMT Val^158^Met polymorphism impacts directly on the level of prefrontal dopamine, these studies support the purported role of dopamine in facilitating EF in infancy.

More recent studies have, however, indicated that the relationship between COMT Val^158^Met genotypes and EF during early childhood may be more complex. In a recent fNIRS study, Moriguchi and Shinohara (2018) found an effect of the COMT Val^158^Met polymorphism in the opposite direction to most previous studies. Japanese children aged 3–6 years who were Val homozygous performed *better* on a task of cognitive shifting, and demonstrated increased activation in lateral prefrontal regions during this task, compared to children with at least one Met-allele (Met/Met and Val/Met). Similar findings, but in a different context, come from a longitudinal study by Blair et al. (2015) that investigated the interaction between early adversity and COMT Val^158^Met genotype in predicting EF in early childhood (7 months – 5 years). Findings from this study indicated that the Val variant was associated with higher EF abilities in children aged 2–5 years. Importantly, it was found that early adversity accounted for faster growth of EF abilities (between ages 3 and 5 years) for children with two Val alleles, compared to children with other COMT Val^158^Met genotypes.

In addition to highlighting the contradictory findings in this area of research and the need for replication, the findings of Moriguchi and Shinohara, 2018, Blair et al., 2015 demonstrate the importance of taking into account (1) potentially different effects in different ethnic groups/populations, as well as (2) gene-environment interaction effects on developmental outcomes. It is well-established that allele frequencies differ substantially between different populations, for example the Met/Met genotype is relatively rare in the Japanese population (Palmatier, Kang, & Kidd, 1999) and functions within the context of a different genetic background. This could result in differential effects of specific polymorphisms such as the COMT Val^158^Met. Furthermore, an extensive literature suggests that certain genotypes may lead to differential, and even opposite effects, depending on which type of environment a child grows up in (e.g., Belsky and van IJzendoorn, 2017, Keers and Pluess, 2017).

Another consideration in understanding the potential impact of dopaminergic genes on the PFC during development is gene methylation, essentially silencing of a gene (Szyf & Bick, 2013). Gene methylation falls under an umbrella of biological interactions at the interface between the genetic code and the environment, referred to as epigenetics. Epigenetics encompasses changes in gene expression caused by biological mechanisms other than the DNA sequence itself (for review, see, Mazzio & Soliman, 2012, and McGowan & Roth, 2015). DNA methylation plays an important role in diversifying genome function during embryonic development, and it was initially believed that these methylation patterns then remained fixed throughout life; however, it is now believed that environmental influences during post-natal development (especially early development) may also have an impact on methylation patterns, which may in turn result in different psychological and behavioural outcomes (McGowan and Roth, 2015, Szyf and Bick, 2013).

A recent study by Lewis et al. (2019) examined the role of non-shared environmental experiences on methylation of dopaminergic genes, and the related association with cognitive task performance, in a cohort of 8 year old identical twins, thus controlling for genetic and early familial influences. They found that twin differences in DNA methylation levels of five dopaminergic genes (including COMT) predicted differences in EF. Interestingly, methylation differences in COMT, DBH (another gene coding for an enzyme which breaks down prefrontal dopamine), the dopamine transporter gene (DAT1) and two dopamine receptor genes (DRD1 and DRD2) were associated with twin differences in inhibitory control, whereas methylation of the dopamine receptor 4 (DRD4) gene was associated with twin differences in short-term memory. This suggests at least some dissociability of dopaminergic gene methylation effects on EF, and further indicates that the environment does indeed have substantial effects on EF during development. Furthermore, a study in adult COMT Val/Val carriers found that lower stress levels were associated with higher methylation of the gene, which in turn resulted in lower COMT activity (essentially a more ‘Met-like’ activity level associated with higher dopamine availability in the PFC) and higher WM performance and associated PFC efficiency (Ursini et al., 2011). As such, epigenetic research may be able to reconcile findings from standard candidate gene studies investigating the effect of variation in genes such as COMT (e.g., Holmboe et al., 2010, Markant et al., 2014) and studies finding contradictory effects in high-adversity samples that are likely to have substantially different environmental exposures (Blair et al., 2015).

## Frontal connectivity: from diversity to specialisation

As part of its process of maturation, the PFC develops rich connections within itself and with other cortical, subcortical and limbic brain regions (Olson & Luciana, 2008), which together form a system that sub-serves EF (Aron, 2008, Casey et al., 2006, Gazzaley and D’Esposito, 2007). The connectivity between the PFC (particularly the DL-PFC) and other brain regions, such as the ACC and the parietal cortex, was recognised by Diamond (2002) as being important for the developmental changes seen during the first year of life; she specifically stated that the PFC cannot work in isolation (p. 494). Since limited research on the development of such functional connections between the PFC and other regions existed at the time, this led Diamond to ask which roles these connections might sub-serve in terms of executive functioning. In this section, we will review the substantial advances that have been made in understanding the interconnected neural system that mediates EF and the functional changes in this system with development.

### Functional connectivity

Functional connectivity between the frontal and parietal cortices has been demonstrated to mediate early EF development in a body of neuroimaging work by Buss and Spencer, 2014, Buss and Spencer, 2018 that utilised fNIRS. In their 2014 study, Buss, Fox, Boas and Spencer confirmed that a fronto-parietal network underlies visual working memory and that, as children age, a greater recruitment of the parietal cortex supports the development of their visual WM capacity. In a later study using the DCCS task (a measure of switching/cognitive flexibility), Buss and Spencer (2018) presented evidence that 3 and 4 year old children displayed different posterior cortical activation on ‘easy’ compared to ‘hard’ versions of the task. A sub-group of 3 year old children who failed the ‘hard’ task showed weaker frontal activation, despite those same children showing strong activation when correctly switching in the ‘easy’ task. Buss and Spencer (2018) explained these results by employing a model known as the dynamic neural field model (Buss & Spencer, 2014). The authors argued that, whilst at age 3 years children have weak neural interactions within the frontal cortex and unrefined fronto-parietal connectivity, by 4½ years children have formed stronger neural connections and developed a refined fronto-parietal pathway with efficient connectivity (Buss & Spencer, 2014; see also, Perone et al., 2015, Perone et al., 2018). This argument is further supported by fMRI research which demonstrated that, compared to adults, children displayed weaker functional connectivity between the lateral PFC, ACC, inferior parietal cortex and ventral tegmental area during the DCCS task (Ezekiel, Bosma, & Morton, 2013). Similar to Buss and Spencer, 2018, Ezekiel et al., 2013 proposed that the development of cognitive switching is underpinned by the development of strengthened connections that make up a cognitive control network, rather than the functional maturation of the lateral PFC alone (Ezekiel et al., 2013, p. 47). Therefore, this body of evidence suggests that developmental improvements in EF task performance are likely due to functional integration via connectivity, just as much as they are due to the architectural and physiological development within the PFC itself (see section on “The development of the PFC”).

Further evidence for the importance of fronto-parietal functional connectivity early in development has been presented by Alcauter et al. (2014), who employed fMRI techniques to establish that activation synchrony among prefrontal and parietal regions, along with connectivity to the thalamus and the salience network (insula, cingulate, frontal cortices) at 1 year of age, was predictive of verbal WM performance at 2 years. The contribution of fronto-parietal functional connectivity has been shown to continue into early childhood. The fronto-parietal network is sensitive to WM load from early childhood (Buss et al., 2014, Perlman et al., 2016), and functional specialisation, including lateralisation, has been shown to continue well into middle childhood (Bell and Wolfe, 2007, Crone et al., 2006, O’Hare et al., 2008, Tsujii et al., 2009, Tsujimoto et al., 2004). For example, fMRI research conducted by O’Hare et al. (2008) indicated that connectivity between the frontal and parietal cortices strengthened with age from childhood (aged 7), through adolescence and into adulthood (aged 28). A linear, load-dependent increase in activation of these areas was found in adult participants. However, in 7 year olds, only the left ventral PFC was activated in response to increasing WM load. Thus, whilst at 7 years activation in the left ventral PFC was mature, the full fronto-parietal connectivity found in adults was not yet fully in place (O’Hare et al., 2008). This provides evidence that refinement and consolidation of this network continues also later in development.

Similar results have been reported within the EF domain of inhibitory control. fNIRS work by Mehnert et al. (2013) revealed age-related inhibitory control improvements accompanied by fronto-parietal functional connectivity differences between 4–6 year old children and adults. Functional connectivity analyses revealed stronger short-range connectivity in the right frontal and right parietal cortices in children during task performance. Adults, on the other hand, had stronger long-range functional connectivity between bilateral PFC and the right parietal cortex than children. These findings suggest that long-range connectivity and specialisation in adults are direct contributors to their superior IC skills. Mehnert et al. (2013) research therefore further bolsters the evidence for the contribution of functional neural connectivity to EF task performance.

Research in recent years has also identified associations between the integrity of fronto-striatal connections and EF performance. Indeed, age-related changes in fronto-striatal functional connectivity have been associated with improvements in cognitive control (Rubia et al., 2006, van den Bos et al., 2015). Research by Achterberg, Peper, van Duijvenvoorde, Mandl, and Crone (2016) reported that in 8–26 year old participants, functional fronto-striatal connectivity predicted improvements in delay of gratification skills (a ‘hot’ measure of IC). This is consistent with previous fMRI research which found that activation in fronto-striatal circuitry correlated with both age and ‘cool’ IC performance in children aged 8–10 years (Durston et al., 2002) (for further discussion of the ‘hot’ and ‘cool’ EF distinction, see section on "Inhibitory control" and Glossary). Finally, research from atypically developing populations, such as children with attention deficit/hyperactivity disorder (ADHD), provides further evidence for the importance of fronto-striatal connectivity in the development of EF by highlighting that impaired EF performance is associated with reduced fronto-striatal connectivity (see Supplementary Box 1).

Electrophysiological research has also offered interesting insights into the role of frontal connectivity. In adults, it has been found that the theta/beta ratio is related to a range of EF processes (Putman et al., 2010, Schutte et al., 2017, Schutter and Van Honk, 2005). Putman, Verkuil, Arias-Garcia, Pantazi, and van Schie (2014) interpreted these results as fitting a framework similar to that of Buss and Spencer's (2014) dynamic neural field model. In their framework, Putman et al. (2014) suggest that a high theta/beta ratio is indicative of poor top-down regulation, which is mediated by the PFC within the fronto-parietal network. Perone et al. (2018) used EEG to investigate how age-related changes in the theta/beta ratio were associated with EF abilities in children aged 3–9 years; in accordance with Putman et al., 2014, Perone et al., 2018 hypothesised that there would be a negative relationship between theta/beta ratio and EF task performance in childhood. This hypothesis was confirmed: using the Minnesota EF scale, they found that individual differences in the theta/beta ratio in both frontal and parietal regions were inversely related to children’s EF performance. According to Putman et al.'s (2014) theory, this may suggest that EF task performance is poorer when top-down processes involving fronto-parietal connections are weak, such as in young children.

### From global to local brain activation: A refinement and specialisation process

In addition to substantial improvements in connectivity between the PFC and other brain regions across development, much of the literature in this area reports a shift with age from global to local activation in the PFC during EF tasks. This shift may signal the increased efficiency of the developing brain and the growing functionality of sub-regions of the PFC for EF (for a review based on early fMRI research, see, Durston & Casey, 2006). An EEG study by Bell and Wolfe (2007) found that 8 month old infants who demonstrated correct A-not-B task performance displayed an increase in global cortical activity but, by 4½ years of age, successful performance on the Day/Night task (a measure of IC) was associated with an increase in localised activity only over the medial-frontal regions. Bell and Wolfe (2007) suggested that this pattern was reflective of more efficient processing as children shift from global cortical activation to more specialised frontal cortex involvement during EF tasks. A body of fMRI research from Durston et al., 2002, Durston et al., 2003, Durston et al., 2006 has further demonstrated this shift from global to local activation during inhibitory control tasks as the PFC matures. Compared to adults, children (6–10 years) recruited larger, more diffuse prefrontal regions during the Go/No-Go task. Children also maximally activated the ventrolateral PFC and the posterior parietal cortex during the task, whereas adults showed a more localised increase in activation in the ventral PFC and in the striatum, suggesting that children needed to engage in compensatory neural activation to achieve the same level of inhibitory performance.

Neuroimaging work on WM development has demonstrated a similar process of refinement and specialisation in frontal regions. Longitudinal fNIRS research by Tsujii et al. (2009) demonstrated that, whilst 5 year old children displayed bilateral activation in the frontal lobe during a WM task, this activation was refined to right frontal regions in 7 year old children. Similarly, fMRI research by Scherf et al. (2006) found that, during two WM tasks, adults recruited a network of localised brain regions that included the left DLPFC, ventrolateral PFC and supramarginal gyrus. Adolescents (14–17 years) however, relied on a more diffuse network that included the DL-PFC, anterior cingulate, posterior parietal cortices, and the anterior insula (Scherf et al., 2006). In comparison, children (10–13 years) relied on ventromedial regions, such as the caudate nucleus and anterior insula, as well as the striatum and cerebellum during WM tasks. Finally, a large decrease in caudate and thalamus activation was seen from childhood to adolescence (Scherf et al., 2006). In sum, this evidence indicates a refinement process in frontal regions starting in childhood and continuing across adolescence and into adulthood.

Finally, this refinement process has also been demonstrated during tasks of cognitive shifting. fMRI research by Rubia et al. (2006) demonstrated that children, but not adults, recruited the DL-PFC during shifting. The authors interpreted this as reflecting compensatory neural activity in the developing PFC. Similarly, recent fNIRS work by Chevalier, Jackson, Roux, Moriguchi, and Auyeung (2019), using a task with both inhibitory and switching demands, identified that less activation in the PFC was associated with better performance in older children (8–9 years), whereas younger children (5–6 years) displayed more reliance on strong PFC activation for successful cognitive control. Chevalier et al. (2019) interpreted these results as evidence that less activation in the PFC in older children may reflect the greater functional connectivity between frontal and parietal regions, leading to increasingly flexible and automatized cognitive control during childhood. This was also the conclusion of Quiñones-Camacho et al. (2019), who similarly found evidence of reduced DL-PFC activation during a cognitive flexibility task in 4–5 year old children who had strong attentional control skills, although in this study there was no direct association between task performance and DL-PFC activation (perhaps due to the younger age of the participants).

**Fig. 4:**
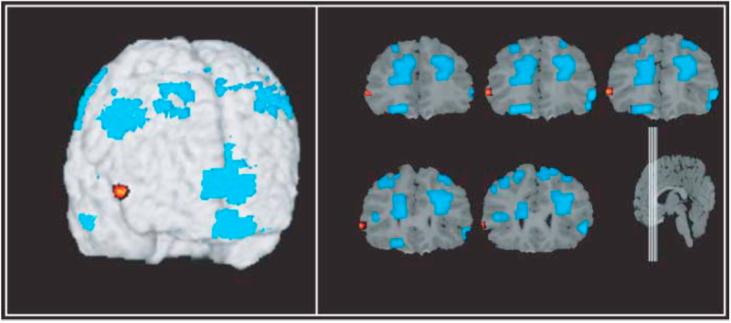
Figure taken from Durston et al. (2006), as published in *Developmental Science, 9 (1)*, and reprinted with permission from the publisher (*John Wiley and Sons*). A longitudinal whole-brain, voxel-based comparison image demonstrating increased activation in the right inferior frontal gyrus (in red/orange) between time 1 (9 years) to time 2 (11 years). Reduced or unchanged activation (in blue) was found in other regions of the prefrontal cortex.

In summary, based on recent fMRI research, it has been suggested that mature EF depends on the focalisation of activity to brain regions directly linked to the cognitive function in question, as well as decreased activity in supplementary brain regions (Durston and Casey, 2006, Durston et al., 2006, Durston et al., 2002, Durston et al., 2003). It should however be noted that many of the fMRI studies carried out during the previous decade had very small sample sizes, often comparing ∼10 children/adolescents (varying widely in age) to ∼10 adults. One exception is a study by Rubia et al. (2007), which investigated the neural correlates of response inhibition using the Stop-signal task in a well-powered sample of adolescents and adults. Here it was found that, in contrast to many previous fMRI studies, there were linear *increases* in inhibition-related brain areas, such as the right inferior frontal cortex (rIFC), with age. Durston et al. (2006), in a small longitudinal study, also found an increase specifically in the rIFC with age, on the background of general decreases in other areas of the PFC (see Fig. 4). These results suggest that developmental changes in the neural substrate of EF may be more nuanced than the diffuse-to-focal view proposes, with some areas decreasing in activation and others increasing in activation with specialisation. This point is also backed up by the recent well-powered fNIRS study by Chevalier et al. (2019), which found that older children were able to engage EF in a more flexible manner (i.e., adjusting cognitive control to specific contexts) than younger children, and that this flexibility impacted on which frontal regions showed increased or decreased activation. More broadly, given the recent failure to replicate many behavioural findings in psychology (Open Science Collaboration, 2015), the field would benefit from replication of some of the early fMRI findings in a larger sample spanning mid-childhood to adulthood, ideally using longitudinal methodology to decrease confounds associated with between-subjects brain and performance variability.

## Conclusion

The closing remark in Diamond's (2002) review says that ‘now’ is an exciting time in frontal lobe research as, finally, the tools exist that enable answers to many of the unanswered questions to be found and, in keeping, this review will end on a similar note. The ‘tools’ Diamond was referring to in 2002 encompassed multiple advances in neuroimaging techniques, advances that have continued to pave the way in developmental cognitive neuroscience in the last two decades. In fact, the use of neuroimaging methods such as fMRI in developmental psychology only began in the 1990s, just a decade prior to Diamond's (2002) review.

Since then, the field has seen a dramatic increase in the way in which neuroimaging techniques are used to provide greater insight into the neural underpinnings of cognitive functioning than was ever possible before. In more recent years, the introduction of fNIRS alongside fMRI and EEG in the developmental researcher’s toolkit has seen the field make substantial advancements, particularly with regards to research into the neural correlates of EF in infancy and early childhood. Similarly, the increasing use of converging methods, as well as longitudinal research, to investigate both neural and cognitive performance trajectories in EF has resulted in new insights into the way in which EF is associated with brain function and development. Perhaps the biggest change that has resulted from this explosion in research since Diamond's (2002) chapter, and one that she foresaw, is the regular identification of developing *networks* of brain areas mediating EF across domains (inhibitory control, working memory and shifting, but also attentional control and similar domains). As such, although the prefrontal cortex undoubtedly still plays a major role in EF across development, it has to be considered within the context of a constant interplay with other key nodes in the network. The last nearly two decades have shown us that the brain networks mediating EF start to become active and functional already in the first year of life, but change substantially throughout childhood and adolescence, both in terms of connectivity and functional specialisation.

Although recent neuroimaging studies have begun to paint a picture of the way in which neural networks change through infancy and childhood, and on into adulthood, there is still more work to be done. fNIRS holds great promise in facilitating the investigation of the neural substrates of EF during the first two years of life, both in terms of localisation of function and functional connectivity. Similarly, whilst advances in structural neuroimaging have enabled a deeper understanding of the contribution to improvements in EF of changes in cortical thickness, volume, and white matter in the PFC and connected areas, future research is needed to identify a detailed neural timeline of structural changes occurring in the PFC from early infancy to adolescence. Novel methods, such as the myelin-specific MRI technique developed by Deoni et al. (2011), will help establish links between specific aspects of PFC maturation and cognitive outcomes. Longitudinal research that involves tracking of both neural and behavioural changes over time will be particularly useful in this endeavour.

Where previously research with infants and toddlers faced difficulties arising from a lack of appropriate tasks for use with infants, the field is now working to overcome this barrier with the invention and increasing use of age-appropriate tasks to measure EF (Conejero et al., 2016, Holmboe et al., 2008, Holmboe, Larkman, et al., 2018, Kovács and Mehler, 2009, Mulder et al., 2014, Wass et al., 2011). Since the overwhelming evidence points to an important period in both maturation of the PFC and EF development in infancy and the pre-school years, it is clear there is excellent value in investigating this area further.

To conclude then, we acknowledge the influential review by Diamond (2002) by reflecting that now, 17 years on, is still an exciting time in developmental executive function research. *Now*, more than ever, the necessary tools exist that may just enable researchers to unlock many of the answers to the questions that Diamond (2002) stated to be of such importance – since, after all, we agree with Diamond (2002) that it is the executive functions and their instantiation in the brain that are responsible for so much of what makes us proud to be human.

## Funding

Karla Holmboe is supported by a Career Development Award from the UK Medical Research Council (Fellowship MR/N008626/1).

## Declaration of Competing Interest

None.

## Glossary

Allele: a variant form of a gene; humans have two alleles on each gene as we inherit one allele from our mother and one allele from our father
Attention deficit/hyperactivity disorder (ADHD): a neurodevelopmental disorder that is characterised by problems with inattention, hyperactivity and impulsivity
Cognitive conflict: processing of conflicting information, which requires cognitive effort to be resolved
Conflict resolution task: a task that places conflicting demands on the participant for them to resolve (e.g., the Simon task and spatial conflict task)
Cool EF: top-down control processes that operate in affectively neutral contexts (e.g., inhibiting a prepotent response in a Go/No-Go task or maintaining neutral information in working memory); can be considered the more cognitive aspects of EF.
Delay of gratification task: a task where the participant (typically a young child) can choose to have a small reward now or a large reward later. Choosing to wait for the larger reward is considered to indicate the ability to delay gratification.
Dendritic trees: branched extension of a nerve cell that integrates inputs from synapses
Dimensional change card sort task (DCCS): a measure of cognitive shifting widely used with children; in pre-switch trials participants must sort cards based on one dimension (e.g., colour), whilst in post-switch trials, they must sort them based on a second dimension (e.g., shape)
Epigenetics: the study of modifications of gene expression
Electroencephalogram (EEG): a physiological technique used to measure electrical activity of groups of neurons in the brain
Event-related potential (ERP): a measure of neuronal electrical activity (obtained using EEG) in response to a sensory, cognitive or motor stimulus or event
Functional connectivity: co-activation between brain regions during rest or task
Functional magnetic resonance imaging (fMRI): a neuroimaging technique which detects changes in blood flow as a result of brain activation, often in response to task stimuli
Functional near-infrared spectroscopy (fNIRS): a neuroimaging technique that uses near-infrared light to detect changes in concentrations of oxygenated and deoxygenated haemoglobin in the brain as an indicator of brain activation
Genotype: the specific combination of alleles possessed by an individual
Go/No-Go task: a behavioural measure of inhibitory control; participants have to respond as fast as possible to a frequently presented target, but withhold their response when a rarer ‘No-Go’ stimulus is presented. Performance is often indexed by the frequency of commission errors/false alarms, i.e., the number or proportion of failed No-Go trials.
Grey matter: part of the brain tissue that predominantly contains neuronal cell bodies
Heterozygous/heterozygote: a genotype consisting of two different alleles (e.g., Val/Met in the COMT gene)
Histological studies: a type of post-mortem examination; the study of microscopic biological material (e.g., brain tissue)
Homozygous/homozygote: a genotype consisting of two copies of the same allele (e.g., Met/Met in the COMT gene)
Hot EF: top-down control processes that operate in motivationally and emotionally significant contexts (e.g., ‘delay of gratification’ tasks where children have to wait in order to receive a reward); can be considered the more emotional/self-regulatory aspects of EF.
Methylation: the addition of a methyl group to DNA that modifies the function of genes by affecting gene expression (in most cases dampening the expression)
Minnesota EF scale: a computerised, standardised assessment of EF skills for use with children aged 2–6 years
Myelination: the formation of a myelin sheath around a neuronal axon (nerve fibre); increases speed of transmission
Neuron proliferation: rapid increase in neurons
Object retrieval task: a behavioural EF task used with infants and young children; participants must attempt to retrieve an object from either an opaque or transparent box or barrier
Phenylketonuria (PKU): a genetic condition whereby individuals are unable to metabolise phenylalanine, which, if not properly managed, can lead to lower levels of dopamine in the prefrontal cortex
Polymorphism: a small genetic variation
Positron emission tomography (PET): a functional neuroimaging technique that uses radioactive tracers to measure blood flow, oxygen use, glucose use, and other metabolic processes
Saccadic reaction time (SRT): speed of an eye movement
Stop-signal task: a behavioural measure of inhibitory control; participants must inhibit a planned motor response when they hear an auditory tone
Structural connectivity: neural pathways between brain regions
Structural magnetic resonance imaging (MRI): a neuroimaging technique; examines the anatomy and physical structure of the brain, e.g., cortical volume and thickness and volumes of grey and white matter
Synapse: a structure in the nervous system that enables the transmission of signals between neurons
Synaptic pruning: the selective elimination of synapses in order to improve efficiency of neural transmission
Synaptogenesis: the formation of synapses between neurons
Theta/beta ratio: the ratio between two different resting state brain rhythms: slow (theta) and fast (beta) frequencies, as measured by electroencephalogram (EEG)
White matter: brain tissue that is predominantly composed of nerve fibres (axons)

## Supplementary material

Supplementary Box 1

## Footnotes

1 See glossary for definitions of underlined words
