# Supplementary material for: Neural substrates of early executive function development
Source: Dev Rev. 2019 Jun;52:42–62. doi: 10.1016/j.dr.2019.100866 (PMC6686207; doi:10.1016/j.dr.2019.100866)
Supplement: Supplementary Box 1 [file mmc1.docx]

**Supplementary Box 1: ADHD and fronto-striatal connectivity**

An important area of research involving children with attention-deficit/hyperactivity disorder (ADHD), who often experience deficits in elements of EF, in particular inhibitory control, offers a perspective into the importance of fronto-striatal connections in the development of EF. Neuroimaging studies have established that, compared to typically developing individuals, children and adults with ADHD have reduced white matter connectivity in fronto-striatal, cingulate and fronto-parietal tracts (Ashtari et al., 2005; Davenport, Karatekin, White, & Lim, 2010; Konrad & Eickhoff, 2010). This reduced connectivity may be associated with the structural abnormalities in frontal, striatal, parietal and cerebellar regions observed in children with ADHD compared to healthy controls (Castellanos et al., 2002; Shaw et al., 2007), and may indicate a maturational delay. These structural differences also contribute to functional impairments that result in deficits in EF task performance. For example, research by Durston et al. (2003) highlights that children with ADHD demonstrate a delay in the fronto-striatal maturation process and experience difficulty in exerting inhibitory control during ‘No-Go’ trials on a Go/No-Go task, failing to recruit the basal ganglia to the same extent as their typically developing peers. Furthermore, research by Shaw et al. (2014) found atypical connectivity between the ventral striatum (part of the basal ganglia) and the orbitofrontal cortex, as well as structural abnormalities in the striatum, in patients with ADHD. These abnormalities likely contribute to problems in planning and inhibitory control, which are key features of ADHD. This research into the ADHD population therefore offers evidence for the importance of fronto-striatal connectivity and the maturation of striatal structures, such as the basal ganglia, also in the typical development of EF.

Please refer to this supplementary material as:

Fiske, A., & Holmboe, K. (2019). Supplementary Box 1: ADHD and fronto-striatal connectivity. Retrieved from <https://doi.org/10.1016/j.dr.2019.100866>.

**References**

Ashtari, M., Kumra, S., Bhaskar, S. L., Clarke, T., Thaden, E., Cervellione, K. L., . . . Milanaik, R. (2005). Attention-deficit/hyperactivity disorder: A preliminary diffusion tensor imaging study. *Biological Psychiatry, 57*(5), 448-455. doi:10.1016/j.biopsych.2004.11.047

Castellanos, F. X., Lee, P. P., Sharp, W., Jeffries, N. O., Greenstein, D. K., Clasen, L. S., . . . Walter, J. M. (2002). Developmental trajectories of brain volume abnormalities in children and adolescents with attention-deficit/hyperactivity disorder. *JAMA, 288*(14), 1740-1748. doi:10.1001/jama.288.14.1740

Davenport, N. D., Karatekin, C., White, T., & Lim, K. O. (2010). Differential fractional anisotropy abnormalities in adolescents with ADHD or schizophrenia. *Psychiatry Research, 181*(3), 193-198. doi:10.1016/j.pscychresns.2009.10.012

Durston, S., Tottenham, N. T., Thomas, K. M., Davidson, M. C., Eigsti, I. M., Yang, Y., . . . Casey, B. J. (2003). Differential patterns of striatal activation in young children with and without ADHD. *Biological Psychiatry, 53*(10), 871-878. doi:10.1016/S0006-3223(02)01904-2

Konrad, K., & Eickhoff, S. B. (2010). Is the ADHD brain wired differently? A review on structural and functional connectivity in attention deficit hyperactivity disorder. *Human Brain Mapping, 31*(6), 904-916. doi:10.1002/hbm.21058

Shaw, P., De Rossi, P., Watson, B., Wharton, A., Greenstein, D., Raznahan, A., . . . Chakravarty, M. M. (2014). Mapping the development of the basal ganglia in children with attention-deficit/hyperactivity disorder. *Journal of the American Academy of Child and Adolescent Psychiatry, 53*(7), 780-789. doi:10.1016/j.jaac.2014.05.003

Shaw, P., Eckstrand, K., Sharp, W., Blumenthal, J., Lerch, J. P., Greenstein, D., . . . Rapoport, J. L. (2007). Attention-deficit/hyperactivity disorder is characterized by a delay in cortical maturation. *Proceedings of the National Academy of Sciences of the United States of America, 104*(49), 19649-19654. doi:10.1073/pnas.0707741104
